# Mental Health in Lebanon's Triple-Fold Crisis: The Case of Refugees and Vulnerable Groups in Times of COVID-19

**DOI:** 10.3389/fpubh.2020.589264

**Published:** 2021-01-20

**Authors:** Fouad M. Fouad, Andres Barkil-Oteo, Jasmin Lilian Diab

**Affiliations:** ^1^Faculty of Health Sciences, American University of Beirut, Beirut, Lebanon; ^2^Faculty of Medicine, American University of Beirut, Beirut, Lebanon; ^3^Refugee Health Program, Global Health Institute, American University of Beirut, Beirut, Lebanon

**Keywords:** mental health, COVID−19, refugees, conflict, Lebanon, policy, political economy

## Abstract

Lebanon's management of the COVID-19 pandemic is largely being maneuvered amid the country's escalating triple fold crisis. As the country continues to grapple with political stagnation, a dwindling economy and currency, all while working through an ongoing refugee crisis, mental health in times of Coronavirus in Lebanon remains unaddressed. This piece explores the effects of this triple fold crisis upon the mental health of the country's refugees and most vulnerable groups, and provides room for discussions on the potential benefits of telemental health as an intervention in low-income and conflict settings. Although the implementation of TMH services in Lebanon among vulnerable communities in times of COVID-19 is not a priority, this piece insists it would ultimately fill a substantial mental health gap during the country's ongoing difficult transitory period.

## Introduction: Lebanon's Triple-Fold Crisis

With ~2,775 cases to this date (1), Lebanon has managed (for now) to contain the spread with an initial strict lockdown, and confinement measures amid an already struggling economy (2). Prior to the COVID-19 pandemic, Lebanon was struggling under the weight of political stagnation, a dwindling economy and currency tarnished by corrupt management and leadership, all while managing an ongoing refugee crisis. A viral outbreak such as Coronavirus, ultimately places additional pressure on the already vulnerable country and its population.

The minister of Public Health declared Lebanon in Phase 4 of the outbreak with 40 have reportedly died from the infection (2). Experts in the field are in consistent fear that the health sector, which is already plagued by medical equipment shortages and dwindling foreign currency reserves, will be overwhelmed if a second wave of infections might hit the country (3). In March 2020, Human Rights Watch warned that Lebanon's overwhelming financial crisis is contributing to “the scarcity of medical supplies necessary to fight the outbreak” (4). Lebanon took “early preventive measures,” such as enforced social distancing, to slow the infection rate (5). Nonetheless, with the reopening of the airport, there has been a surge in infection rates, partly driven by expats and the other by the apathy among the public to maintain social distance measures to wear masks. Top health officials are warning about the possibility of having to enforce lockdown measures again^1^, however it remains to be seen whether or not Lebanon is in fact capable of reinforcing this strict level of compliance, particularly if financial support is not provided to the most vulnerable and poor factions of Lebanon's general public. The country would need to provide continued support to the 300,000 families that live below the poverty line in Lebanon as of 2019, with the latest estimates unavailable (6).

A World Bank Report from 2019 expects that 50% of the Lebanese population will fall on, or below the poverty line if the economic crisis is not swiftly addressed (7). The economic crisis combined with the COVID-19 pandemic bring additional strains upon an already-strained population. Since October 2019, the country has grappled with mass protests denouncing the incompetence and corruption of the serving political class as well as their inability to respond justify the country's debt, and address the severe economic recession (8). According to Human Rights Watch, over 220,000 people (9) lost their jobs this year and others are victim to pay cuts and a declining Lebanese pound value, which has lost over 80% of its value on the black market (10). Estimates on unemployment in Lebanon range between 25 and 40% (11).

COVID-19 essentially serves as the country's third level of constraint amid ongoing political and economic challenges. While violence has remained sporadic and scattered more recently, with small clashes and protests erupting across the country since the lockdown has eased, it is only anticipated to increase as the economic situation deteriorates further, and more people struggle to make ends meet. Lebanese political parties are attempting to rebuild their reputation by opening medical centers in their respective regions, but they lack the financial support their sponsors once gave them amid riots breaking out more violently as time passes (12).

## Lebanon's Refugees Are Additionally Vulnerable

In the 9 years since the conflict in Syria began, an estimated 1 million Syrian refugees have sought safety in Lebanon, ultimately making it the largest concentration of refugees per capita in the world (13). Lebanon's deep economic crisis has rendered Syrian refugees more economically vulnerable, with 75% living under the poverty line, according to VASyR^2^. The majority of refugees do not have regular access to basic water, sanitation, hygiene services or infrastructure (14). Absence of permanent solutions to access to water and sanitation structures, such as connection to municipal water and sanitation networks, contribute to their dire situation (14). This increases refugees' risk of exposure to infectious and preventable diseases, as well as other health issues.

When the first case of COVID-19 was diagnosed in Lebanon, citizens were requested to improve their hygiene practices and follow social distancing protocols in order to “flatten the curve” of the virus (15). However, the implementation of these practices in refugee settlements, where water is scarce and people depend on humanitarian organizations for water supplies, proved to be particularly difficult (16). Furthermore, overcrowding and poor sanitation in settlements make physical distancing difficult, exposing refugees to enormous health risks (16). Action Against Hunger adapted their programming to support the severely vulnerable Syrian refugees in informal settlements throughout the crisis, attempting to provide the settlements with as much clean water as possible, but the amount is far from enough to cover all household needs for personal hygiene, disinfection of tents, washing of clothes, cooking, and more (16).

According to the UNHCR, since the beginning Lebanon's economic deterioration more and more Syrian refugees are reportedly resorting to negative measures such as spending less on food (17). One-third of adult refugees restrict their food consumption in order to ensure their children can eat (17), and a reported 3/4 refugees reduce the number of daily meals (17). Several individuals residing in settlements rely mainly on loans and credit purchases from shops and neighbors, but even this has been increasingly impossible (17).

## Obstacles to Mental Health: Stress and Trauma in Conflict Among Refugee Communities

Across the MENA region, the COVID-19 pandemic is taking a significant toll on the mental health and psychosocial well-being of individuals from all factions of society. Among the most vulnerable, are refugees, asylum-seekers, internally displaced and stateless people, all of whom grapple with their own traumas and health concerns amid worrying about their uncertain legal and economic statuses. UNHCR reports that the COVID-19 pandemic has exacerbated old vulnerabilities while steadily increasing the intensity of these realities among vulnerable groups, and triggering a wide range of mental health conditions, which may lead long-term psychosocial consequences' (18). Fear of deportation or eviction, discrimination, as well as loss or reduced livelihoods remain major sources of psychosocial distress according to their report (18). Additionally, these fears have laid the foundation for negative social reactions, including but not limited to: panic, stigma and discrimination in the communities (18). Reports additionally highlight the fact that some of the most vulnerable are exhibiting high levels of psychological distress (18). This includes individuals and families with pre-existing mental health conditions or substance abuse issues, as well as other particular cases the UNHCR highlights as persons of concern like older refugees and refugees with disabilities (18).

Syrian refugees in Lebanon are subject to a number of stressors which are the direct result of traumatic events in conflict situations or the daily hurdles being a refugee. The mere fact of struggling to uphold essential living conditions as well as living through the uncertainty about their legal standing and livelihood are but the tip of the iceberg of everyday challenges for Syrian refugee communities. War-related traumas, coupled with daily stressors have major implications upon the long-term psychosocial well-being of refugees. The UNHCR and its partners state that social distress, cognitive problems, chronic pain, and PTSD are typical documented reactions from prolonged stress situations among refugees in Lebanon (18). The UN agency along with grassroots organizations have insisted that mental health services are necessary to elevate the living standards of displaced Syrians across Lebanon and the region. Despite the need for mental health and psychosocial support (MHPSS) being alarmingly high and options in the region are scarce in the areas of mental health in general. In addition to the lack of supply, challenges exist to accessing the available MHPSS services (18).

Mental health and psychosocial support (MHPSS) activities are being stepped up by the UNHCR and a number of grassroots and international organizations with the aim of addressing this new escalating trend before it is no longer repairable. In Lebanon more specifically, multiple incidents of suicide were reported among refugees in 2020 (19). Refugee settlements across the country have witnessed a hike in instances of threats to self-harm and harm to others while family disputes, domestic violence, and divorce cases have increased (20). Family members and young children are those primarily at risk of domestic violence, in particular women and young girls (20). Unable to pay rent and facing evictions, refugees reportedly resorting to sharing accommodation, further increasing the risk of sexual, and gender-based violence (SGBV) and strained mental health implications (20).

A study conducted in a joint effort by the Danish Refugee Council and partners in 2018 found that there is a large gap between the need of MHPSS among Syrian refugees and quality of services they actually receive. Of the 1,082 Syrian refugee participants who took part in the comprehensive study, 62% expressed that they needed assistance to deal with physical pain and distress (21). Additionally, 55% of the sample suffer from distress and 28.5% feel severely or extremely emotionally affected by their health issues (21). In the areas of “trauma,” 32.5% of respondents reported being exposed to one or multiple traumatic events (21). There traumatic events included loss of a close family member, exposure to violence and/or being arrested, detained or imprisoned (21). Additionally, the study highlighted that those who had lost a close family member, had a much higher risk of experiencing pain and distress, having functionality difficulties in their everyday life and having poor self-rated health (21). Separate factors influencing distress, pain, functionality and self-rated health were age, gender, exposure to violence coupled with unmet basic needs (21). The study further highlighted the strong positive correlations between pains, self-rated health and being emotionally affected by health problems, and established that psychosocial challenges and pain occur simultaneously and reinforce each other (21). The study further touched upon basic needs, such as food, shelter, security, and employment and how they played a fundamental role in the experience of distress (21).

Across Lebanon, refugee outreach volunteers have been able to reach over 4,000 persons with community-based psychosocial support messaging since the beginning of the COVID-19 pandemic (22). Since February, outreach volunteers, community groups and other structures have reached more than 332,000 individuals (over 50% women) through virtual information and awareness sessions, including on general COVID-19 prevention messages, community-based MHPSS, and parenting skills, among others (22). Awareness sessions with some of the most vulnerable groups to reach are being conducted through social media platforms, WhatsApp and Zoom, and follow-up sessions are conducted through phone calls (22). Tele-mental health (TMH) efforts have proven to be a successful route during these difficult periods, as international organizations, UN agencies and their partners continue to provide individual psychosocial support sessions remotely, via voice or video call (22). Psychosocial support sessions are taking place face-to-face only for high-risk urgent cases, including but not limited to refugees with suicide ideation, survivors of SGBV, children in the worst forms of child labor, and persons of concern with pre-existing mental health or psychosocial distress, while taking precautionary measures for the safety of refugees and frontline workers (22).

## Telemental Health as an Intervention in Low-Income Settings

Amidst the need for social distancing as well as prevention measures in the times of COVID-19, a tele-mental health (TMH) approach would be suitable to bridge the mental health gap in vulnerable communities across Lebanon and the region. TMH is a form of telemedicine that provides mental health assessment and treatment at a distance, and can provide valuable assistance in light of extremely limited mental health services on the ground. Several studies have shown that TMH could be effective in the diagnosis, assessments, and treatment of a broad range of clinical conditions, and in different practice settings, often comparable to face-to-face outcomes (23). TMH can be successfully used in clinical work and education initiatives alike, it enables clinical consultations and the possibility of providing training and supervision (24). TMH has been shown to be cost-effective (25) with good clinical outcomes mainly as it increases access to care (26). It is a flexible modality that could be adapted to be used in different settings and types of practice (27).

There are two main modalities of communication technologies in the implementation of TMH. One is “synchronous or interactive” and the other is “asynchronous or store-and-forward.” Synchronous services usually provide a live, two-way interactive connection between a provider and a patient who are in distinct physical locations. This modality is commonly used in providing direct clinical services, it could be seen as a “replacement” of the clinical encounter, however, it requires a reliable internet connection and dedicated available staff. The store-and-forward (S&F) mode of connection takes the form of capturing and then send the clinical information via secured emails/websites for a review by a specialist at a later time. Unlike direct (synchronous) forms of connection, an indirect (asynchronous) connection does not require the presence of both parties at the same time. This modality is often used in providing clinical consultations by an expert (second opinion) to a provider who has clinical questions regarding a clinical condition. The advantage of this model is that could be done over a low or unreliable connection, it is very suitable in doing clinical training and supervision for low skilled staff, however, it can't be used to deliver direct services and requires a healthcare staff who is the primary provider for the patient.

TMH initiatives among refugee settings have been growing over the last years, one initiative with Syrian refugees in Turkey showed that it is feasible and could lead to better clinical outcomes and increase satisfaction among providers and patients (28). In Denmark, TMH was studied among groups of asylum seekers, refugees, and migrants who received services from providers who spoke their language. Patients reported a high level of satisfaction and acceptance of the service and stated that they will advise others to use it (29). In the Lebanese context specifically, it has been recently suggested that TMH could be very helpful to increase access to MH services namely through National primary healthcare centers network as a way to leverage the spread of primary care network in Lebanon with specialists input who are largely concentrated in Beirut (30). However, as the region is ill-equipped in the areas of mental health on a broader level, a move toward tele-mental health brings forth pivotal barriers toward its proper implementation on cultural, technical, financial, and regulatory levels in the Lebanese case specifically (31).

In Lebanon, as interpersonal relationships are valued and direct doctor–patient interaction is expected, barriers to using technology in delivering medical services would typically arise. In Lebanon, as in the region, patients are concerned with their physician's background and culture as well as the privacy during encounters in more sensitive cases such as that of undocumented individuals, or unregistered refugees. In a study conducted by Jefee-Bahloul, Syrian refugees were asked about their acceptance of mental and TMH. Reluctance toward a technological approach presented themselves in the form of concerns for privacy, distrust, distortions to the doctor-patient relationship, and unfamiliarity with the technology itself (31). However, despite these early concerns (as shown by several projects), people were open to seeking mental health services using this modality, and once they do they are generally satisfied by the experience.

Additional obstacles to a successful move toward tele-mental health, is the existing infrastructure barriers in Lebanon, which include electricity and internet access (32). Electricity is a prerequisite for tele-medicine and TMH, and must be coupled with high bandwidth capacity to ensure picture and voice clarity (32). These elements are pivotal to ensure a healthy therapeutic relationship between the patient and their physician (32). The availability of trained technical support personnel and medical support for remote/conflict settings represents another obstacle in areas suffering from a shortage of technical and medical services to begin with (32). Given this limitation, implementing an asynchronous (store & forward) modality for clinical consultations may be much more effective than the synchronous modality.

Funding is another fundamental obstacle in the Lebanese case. Amid the country's ongoing economic crisis, although the private sector may pioneer TMH projects and implement the technology faster than public facilities, sustainability remains an issue. While cost effectiveness of TMH has been established in “developed” states, doubts and question marks about it in the MENA region—and in Lebanon more specifically—do exist. Until more studies demonstrate cost effectiveness of TMH in Lebanon and the region, convincing the government, investors and stakeholders to invest in this process will prove to be difficult and far reaching.

In addition to the financial barriers Lebanon currently faces, the policy frameworks are another. It is often the case that public policy is driven by various approaches and philosophies in the areas of health care, which essentially vary depending on the serving political class and its agenda. This would ultimately vary by region as well. As TMH remains “outside” health systems in the region, there are essentially no concrete guiding regulations or policies to frame such practices. Medico-legal issues, licensing requirements, regulation, and quality assurance issues would naturally arise soon after such implementation—and with the extensive hits to the health system in Lebanon since the Civil War, coupled with the current COVID-19 spread, priorities are most definitely not headed in this direction (32). Additionally, confidentiality, data protection, and patient privacy are essential issues in TMH. Fear of political persecution is a threat unique to, and commonly seen in, the region and Lebanon (33). These fears coupled with the “vague” legal frameworks surrounding the country's refugee community and the internally displaced may impact their acceptance to the technology, as well as open up the debates surrounding policies to protect information transmitted electronically (33). Despite all these challenges, and as seen in other settings, COVID-19 has facilitated an explosion in telemedicine and tele mental health applications, proving that many policy hurdles were largely due to inertia rather than having any specific objections to TMH. One example in Lebanon is the relaxing of prescription guidelines by MOH to accommodate for the medical and psychiatric sessions that took place remotely when patients were not able to come to clinics due to the lockdown. Major crises like COVID-19 or the upcoming political and economic crisis could also prove an opportunity to create models of care out of necessity that was not possible previously.

## Concluding Remarks

The implementation of TMH services in Lebanon among vulnerable communities in times of COVID-19 is not a priority, but would ultimately fill a substantial mental health gap. TMH is a feasible solution to increase access of quality mental health services among vulnerable communities in conflict settings; however, its implementation in Lebanon currently stands in the face of multiple intersectional barriers on the political, economic and health levels. In the past, Lebanon has not expressed its readiness to adopt policies to foster the mental health of these communities as well as the staff that assists them, let alone its readiness to delve into the technological requirements to build TMH systems. Approaches to building these systems should be based on preliminary assessment of the specific cultural, financial, legal, and infrastructural needs of Lebanon—a reality currently hindered by the corruption the system is attempting to overcome amid its dwindling currency. Additionally, overcoming the cultural barriers associated with mental health services in general, and TMH services more specifically, requires strategies for training providers and increasing their exposure to the technology and their awareness of its applicability. In order to be effective, this strategy needs to be coupled with an increasing public awareness campaign on the use and effectiveness of technology in order to facilitate patients' acceptance of services of this nature. Additionally, public consultation in all stages of developing TMH, and ensuring the innovations are contextually appropriate and relevant is essential. As the saying goes: “Find the opportunity that lies in every crisis,” the current crisis in Lebanon will have long-term effect, and requires long term planning. Despite the obstacles, investment in TMH system could help citizens and refugees alike, given the successful implementation of TMH in other low-income settings (34). We do believe that instead of keep trying the same service model repeatedly, maybe it is time to create a new service delivery system that could help Lebanon not just sustain but significantly improve their mental health delivery system.

## Author Contributions

All authors listed have made a substantial, direct and intellectual contribution to the work, and approved it for publication.

## Conflict of Interest

The authors declare that the research was conducted in the absence of any commercial or financial relationships that could be construed as a potential conflict of interest.

## Footnotes

^1^Available online at: https://www.dailystar.com.lb/News/Lebanon-News/2020/Jul-16/509094-two-deaths-and-57-virus-cases-confirmed-total-2599.ashx

^2^Available online at: https://reliefweb.int/report/lebanon/vasyr-2019-vulnerability-assessment-syrian-refugees-lebanon
